# Acaricide resistance status of livestock ticks from East and West Africa and *in vivo* efficacy of acaricides to control them

**DOI:** 10.1016/j.ijpddr.2024.100541

**Published:** 2024-05-06

**Authors:** Alec Evans, Maxime Madder, Josephus Fourie, Lénaïg Halos, Bersissa Kumsa, Elikira Kimbita, Joseph Byaruhanga, Frank Norbert Mwiine, Dennis Muhanguzi, Safiou Bienvenu Adehan, Alassane Toure, Jahashi Nzalawahe, Fred Aboagye-Antwi, Ndudim Isaac Ogo, Leon Meyer, Frans Jongejan, Imad Bouzaidi Cheikhi, Maggie Fisher, Peter Holdsworth

**Affiliations:** a Clinglobal, B03/04, The Tamarin Commercial Hub, Jacaranda Avenue, Tamarin, 90903, Mauritius; b Clinvet USA, 1479 Talmadge Hill South, Waverly, NY, 14892, USA; c Bill & Melinda Gates Foundation, Seattle, WA, USA; d Department of Parasitology, College of Veterinary Medicine and Agriculture, Addis Ababa University, Bishoftu, Ethiopia; e Department of Veterinary Microbiology and Parasitology, College of Veterinary Medicine and Biomedical Sciences, Sokoine University of Agriculture, PO Box 3019, Morogoro, Tanzania; f Department of Bio-molecular Resources and Bio-laboratory Sciences (BBS), College of Veterinary Medicine, Makerere University, Kampala, Uganda; g National Institute of Agricultural Research (INRAB), Animal and Fisheries Health and Nutrition Support Laboratory (LASNAH), 01 BP 884, Cotonou, Benin; h Université Nangui Abrogoua, UFR Sciences de la Nature, 02 Bp 801 Abidjan 02, Côte d’Ivoire, Nigeria; i Department of Veterinary Microbiology, Parasitology and Biotechnology, College of Veterinary Medicine and Biomedical Sciences, Sokoine University of Agriculture, Morogoro, Tanzania; j Department of Animal Biology and Conservation Science, School of Biological Sciences, College of Basic and Applied Sciences, University of Ghana, Legon-Accra, Ghana; k National Veterinary Research Institute, Vom, Plateau State, Nigeria; l Clinvet S.A., Douar Dbabej, Beni Yekhlef B.P 301, CP 28815, Mohammedia, Morocco; m Department of Veterinary Tropical Diseases, Faculty of Veterinary Science, University of Pretoria, Onderstepoort, 0110, South Africa; n Veterinary Research Management Limited, Shernacre Cottage, Lower Howsell Road, Malvern, WR141UX, Worcestershire, United Kingdom; o PAH Consultancy Pty Ltd, 3/27 Gaunson Crescent, Wanniassa, Canberra, ACT, 2903, Australia

**Keywords:** Acaricide resistance, Efficacy, *Rhipicephalus microplus*, *Rhipicephalus appendiculatus*, *Amblyomma variegatum*, Ruminant

## Abstract

Through a collaborative effort across six Sub-Saharan African countries, using recognized international assessment techniques, 23 stocks of three tick species (*Rhipicephalus microplus, Rhipicephalus appendiculatus* and *Amblyomma variegatum)* of economic importance for rural small holder farming communities from East and West Africa were collected from cattle*,* and evaluated in *in vitro* larval packet tests (LPT). The results demonstrated medium to high resistance to chlorfenvinphos and amitraz across species. *Rhipicephalus microplus* demonstrated high level alpha-cypermethrin and cypermethrin resistance. Stocks of *A*. *variegatum* (West Africa) and *R*. *appendiculatus* (Uganda) demonstrated medium level ivermectin resistance.

The four least susceptible stocks (East and West African *R. microplus, A. variegatum* and *R. appendiculatus)* were taken into *in vivo* controlled cattle studies where fipronil was found effective against West and East African *R. microplus* isolates although persistent efficacy failed to reach 90%. Cymiazole and cypermethrin, and ivermectin based acaricides were partially effective against *R. microplus* without persistent efficacy. Flumethrin spray-on killed *A. variegatum* within 72 h for up to 10 days posttreatment, however product application was directly to tick attachment sites, which may be impractical under field conditions. A flumethrin pour-on formulation on goats provided persistent efficacy against *A. variegatum* for up to one-month. Therapeutic control was achieved against *R. appendiculatus* through weekly spraying cattle with flumethrin, amitraz or combined cymiazole and cypermethrin. A fipronil pour-on product offered four-week residual control against *R. appendiculatus* (with slow onset of action).

Few studies have assessed and directly compared acaricidal activity *in vitro* and *in vivo.* There was some discordance between efficacy indicated by LPT and *in vivo* results. This observation calls for more research into accurate and affordable assessment methods for acaricide resistance.

No single active or product was effective against all three tick species, emphasising the need for the development of alternative integrated tick management solutions.

## Highlights

• 23 tick stocks originating from six Sub-Saharan countries were challenged with acaricides representing all currently available classes in Larval Packet Tests.
• Mono- and combination commercial acaricide formulations were evaluated against the most resistant tick stocks *in vivo*.
• Fipronil controlled West/East African *R. microplus* and *R. appendiculatus* isolates.
• Amidine and synthetic pyrethroid sprays provided therapeutic efficacy for *A. variegatum*.
• No single acaricide demonstrated acceptable efficacy against targeted tick species.

## Graphical abstract

**Figure:**
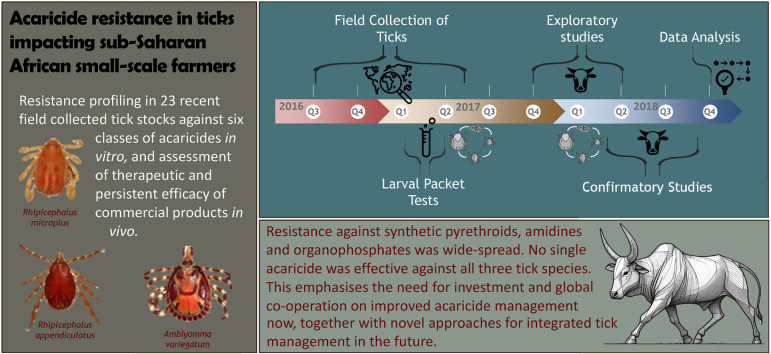

## 1 Introduction

Tick control is a critical part of cattle and small ruminant husbandry in much of Sub-Saharan Africa. Many of the current acaricides have been in use for decades and treatment failure poses a major threat to livestock farmers in the region. Several factors may contribute to the establishment and spread of acaricide resistance in field populations of ticks. Among these are the methods used to apply acaricides to livestock (Kamidi and Kamidi 2005; Vudriko et al., 2016, 2018; Nagagi et al., 2020) and their misuse (Mutavi et al., 2021) which is associated with underdosing and selection pressure for acaricide resistance in tick populations (Githaka et al., 2022). Moreover, variable quality of veterinary medicines in this region has been identified as an issue, with products sometimes not containing the concentration of active ingredient stated on the label (Mouiche et al., 2019).

Recently, the dispersal of *Rhipicephalus microplus* in Sub-Saharan Africa and the adoption of exotic cattle breeds to support the intensification of the dairy industry, have been identified as factors likely to increase acaricide resistance in the region (Githaka et al., 2022) with a wide range of specific interventions tabled to deal with this (e.g. Abbas et al., 2014).

Acaricide resistance monitoring of tick populations at national or regional levels is warranted as part of a tick management program with aims, among others, of identifying and delaying development of resistance (Githaka et al., 2022). Early detection and intervention are of particular importance in achieving this (Vudriko et al., 2016). The recommended approach is that cases of suspected in field-tick acaricide resistance should be confirmed using laboratory based *in vitro* assays such as the larval packet test (LPT) (Food and Agricultural Organization 1984); the larval immersion test (Shaw 1966) or the adult immersion test (Drummond et al., 1973) and the ensuing results correlated against known management practices (Sun et al., 2011). Harmonizing the usage of, and training with, the LPT would assist in production of standardized data on the status of tick resistance in the field (Githaka et al., 2022). The recognized value of these tests must be tempered by the fact that, among other things, they are not sensitive enough to detect the emergence of resistance in early stages and that they take time to deliver results (Abbas et al., 2014), as well as the inaccessibility of these techniques to many low resource laboratories. Moreover, the limitations of *in vitro* tests include the inability to evaluate the impact on efficacy of the interactions between acaricide, host and tick, which at present can only be captured in *in vivo* studies.

Although all three species are not present in all countries, the impact of the three main tick species (*R. microplus, Amblyomma variegatum* and *Rhipicephalus appendiculatus*) of economic importance for rural small holder farming communities in various West and East African countries (Burkina Faso, Ghana, Benin, Nigeria, Ethiopia, Uganda and Tanzania) is paramount. Where each of these tick species is endemic, it is generally highly prevalent with substantial populations on each animal (Heylen et al., 2023). A recently published review summarizing acaricide resistance in Africa further reinforces the relevance of focusing on these tick species (Githaka et al., 2022).

Through a collaborative effort across six Sub-Saharan African countries and using international standards for *in vitro* and *in vivo* animal assessments, within the constraints of time and funding, this study was designed to identify and assess, in detail, the efficacy of acaricides against resistant ticks as an indicator of whether current tick control strategies are effective and sustainable. Several tick stocks were isolated in the field in 2016 and 2017 and transferred to research facilities in the Netherlands [Food and Agricultural Organization (FAO) Reference Centre for Ticks and Tick-borne Diseases]. Larvae from engorged females collected in the field were used for initial screening. The acaricide resistance/susceptibility status of the tick stocks was determined through *in vitro* LPT studies and the least susceptible stocks were selected for further evaluation through *in vivo* studies conducted in Morocco, which are reported on here. In order to provide an evidence-based understanding of the current efficacy of commercialized acaricide products used in the field, we aimed to apply the standardized protocols and calculation methodology for acaricide efficacy as developed for veterinary product registration studies (Holdsworth et al., 2006).

## 2 Materials and methods

### 2.1 Tick stocks

Tick stocks evaluated in this program were collected as part of a surveillance study to assess the status of important endemic and invasive ticks of cattle across at least two districts in six Sub-Saharan African countries (West Africa: Ghana, Benin, Nigeria and East Africa: Ethiopia, Uganda, Tanzania), reported on by Heylen et al. (2023). Countries were selected to provide a representative sample from West and East Africa within the time and funding constraints of the project. Districts within each country were selected based on high cattle populations and access to farmers through regional collaborators. Briefly, engorged female ticks were collected from cattle using forceps and stored in plastic vials fitted with a fine mesh to allow for air exchange. Ticks were collected from up to 120 cattle irrespective of gender, if they were one to two years old, had not been treated with a topical or systemic acaricide during the two weeks prior to the sampling visit and were not excessively fractious in that they posed a danger to themselves or study site personnel. The districts were used to define the stock and where several villages within a single district were sampled, the villages were differentiated by a number after the district (i.e. Serere and Serere 1) After collection, ticks were packaged in International Air Transport Association (IATA) compliant packaging and transferred to the FAO Reference Centre for Tick and Tick-borne Diseases at Utrecht University in the Netherlands for LPTs.

#### 2.1.1 Rhipicephalus microplus

*Rhipicephalus microplus* (formerly *Boophilus microplus*) was found in six of the seven countries: in East Africa in Tanzania and Uganda and in West Africa in Burkina Faso, Ghana, Benin and Nigeria. Samples were collected from two separate sites per country (Supplementary Table 1). These stocks were evaluated in a preliminary round of LPTs from which the least susceptible stocks from East and West Africa were identified and compared against a susceptible strain in a second evaluation, to calculate the level of resistance.

#### 2.1.2 Amblyomma variegatum

*Amblyomma variegatum* was found in all surveyed countries and one stock was transferred from each country, (Supplementary Table 1) with the exception of Tanzania where two stocks were isolated**.**

#### 2.1.3 Rhipicephalus appendiculatus

*Rhipicephalus appendiculatus* was only found in two of the three countries sampled in East Africa Tanzania and Uganda. For this species, three stocks were isolated from Uganda and two from Tanzania (Supplementary Table 1).

#### 2.1.4 Acaricides tested

Compounds representing the most common classes of acaricides were selected for the LPTs in order to provide a complete assessment of resistance and are summarised in Table 1.

**Table 1:** Acaricides selected for evaluation in Larval Packet Tests.

Acaricide Class	Active Ingredient^a^	Minimum Concentration (mg/mL)	Maximum Concentration (mg/mL)
Organophosphate	Chlorfenvinphos	0.00003	3
Phenylpyrazole	Fipronil	0.000256	4
Synthetic Pyrethroid	Alpha-cypermethrin	0.00064	10
Formamidine	Amitraz	0.00001	10
Isoxazoline	Fluralaner	0.001458	2
Macrocyclic Lactone	Ivermectin	0.186624	4

a Commercial product names in sequential order: Supadip, Frontline, Sypertix, Norotraz, Bravecto, Ivomec.

For the *in vivo* studies, acaricides were selected based on their expected efficacy, commercial availability and potential importance for small-scale rural farmers in East and West Africa (Table 2).

**Table 2:** Acaricides selected for evaluation in *in vivo* studies.

Acaricide Class	Active Ingredients^a^	Prepared Formulation Concentration, Dose Route (Dose Rate)
Phenylpyrazole	Fipronil 1%	1 mL/10 kg pour-on (10 mg Fipronil/10 kg)
Phenylpyrazole and Macrocyclic Lactone	Fipronil 0.9%, Abamectin 0.5%	1 mL/10 kg pour-on (9 mg Fipronil and 5 mg Abamectin/10 kg)
Synthetic Pyrethroid	Flumethrin 2%	Formulation diluted at 10 mL/5 L (animals were thoroughly wetted)
Synthetic Pyrethroid	Flumethrin 1%	1 mL/10 kg pour-on (10 mg Flumethrin/10 kg)
Benzoylurea and Synthetic Pyrethroid	Fluazuron 2.5%, Flumethrin 1%	1 mL/10 kg pour-on (25 mg Fluazuron and 10 mg/kg Flumethrin/10 kg)
Benzimidazole	Triclabendazole 12%, Oxfendazole 4.53%	1 mL/10 kg oral (120 mg Triclabendazole and 45.3 mg Oxfendazole/10 kg)
Amidine	Amitraz 12.5%	Formulation diluted at 10 mL/5 L (animals were thoroughly wetted)
Macrocyclic Lactone and Salicylanilide	Ivermectin 80 mg/100 mL, Oxyclozanide 6 mg/100 mL	2.5 mL/10 kg oral (2 mg Ivermectin and 0.15 mg Oxyclozanide/10 kg)
Spiroindoles and Macrocyclic Lactone	Derquantel 10 mg/mL, Abamectin 1 mg/mL	2 mL/10 kg oral (20 mg Derquantel and 2 mg Abamectin/10 kg)
Macrocyclic Lactone and Salicylanilide	Ivermectin 0.5%, Closantel 12.5%	1 mL/25 kg injectable (2 mg Ivermectin and 50 mg Abamectin/10 kg)
Formamidine and Synthetic Pyrethroid	Cymiazole 17.5%, Cypermethrin 2.5%	Formulation diluted at 15 mL/10 L (animals were thoroughly wetted)
Organophosphate and Synthetic Pyrethroid	Chlorfenvinphos 30%, Alfamethrin 3%	Formulation diluted at 10 mL/10 L (animals were thoroughly wetted)
Amino-acetonitrile derivatives	Monepantel 25 mg/mL	1 mL/10 kg oral (25 mg/10 kg)

a Commercial product names in sequential order: Actyl, Attila, Bayticol dip and spray, Bayticol pour on, Drastic Deadline Extreme, Flukazole C, Milbitraz, Oravec, Startect, Sudox Double Endectocide, Tickotan, Zeropar Dip and Spray, Zolvix.

### 2.2 *In vitro* studies: larval packet test

This component of the work evaluated the occurrence and scale of acaricide resistance in first generation larvae from field collected adults from the target geographies. Preferably two stocks of each of the three targeted tick species per country were evaluated, in order to identify the least susceptible stock of the respective tick species for use in *in vivo* studies.

The FAO recommended LPT procedure (Food and Agricultural Organization 2004) was utilised to assess susceptibility to six classes of acaricides.

For each acaricide, a seven step dilution series, using one part olive oil and two parts trichloroethylene as the solvent, was set up to obtain a concentration gradient resulting in 0–100% larval mortality with each concentration tested in triplicate. Individual packets were prepared by adding 0.9 mL of the relevant solution to Whatman #1 filter papers of 10 cm × 7.5 cm. Prepared packets were subsequently packaged and distributed to individual testing laboratories. After insertion of approximately 50 tick larvae, the open side of each packet was sealed and the packets were incubated at ∼28 °C and 75–85% relative humidity (RH). After 24 h (48 h for amitraz), the packets were opened, and live and dead larvae were counted. The ability of the larvae to move on the surface of the filter paper was used as the criterion for determining their viability.

To calculate the average mortality, the following formula was employed:

% larval mortality = (Dead larvae/Total number of larvae) x 100

Mean percentage of mortality = (mortality 1 + mortality 2 + mortality 3)/3

As per the FAO guidelines, mortality in the control packets was used to adjust mortality in the treated packets.

If mortality was <5%, the direct mortality figures were used to calculate the LC_50_. If mortality was between 5 and 10%, the percentage of mortality in all the groups was corrected using the Abbott's formula: Corrected percent mortality = 100 x (% test mortality - % control mortality)/(100 - % control mortality). If mortality in the control group was >10%, the test results were considered invalid, and the results were discarded.

To calculate the resistance factor, a laboratory reared susceptible strain was included for *R*. *appendiculatus* and *R*. *microplus*, both originating from the Republic of South Africa. No susceptible strain of *A. variegatum* was available and as such, the level of resistance was estimated by comparison with a susceptible strain of *R. appendiculatus* as a proxy as it is the most comparable three host tick because both species mainly feed as adults on cattle and have alternative hosts for the immature life cycle stages.

### 2.3 *In vivo* studies

#### 2.3.1 Study design

All exploratory and confirmatory studies were parallel group designed, blinded (with the exception of studies 389 and 391), randomized block design, single centre, negative controlled, efficacy studies. All studies occurred between 2017 and 2018. Approval to perform each individual study was obtained from the Clinvet Institutional Animal Care and Use Committee.

#### 2.3.2 Animal selection and husbandry

Animals were included in the study if they had been acclimatised to the study site for at least seven days. They were clinically healthy, not detectably pregnant and they had not been treated with a long acting topical or systemic acaricide/insecticide during the 12 weeks preceding study start. The health of animals was monitored through daily health checks, regular clinical examinations, and body weight assessments. Cattle (*Bos taurus*) and goats (*Capra aegagrus hircus*) were housed in facilities that complied with Directive 2010/63/EU (European Union, 2010).

##### 2.3.2.1 Exploratory studies

Each of four exploratory studies (study 385, 387, 389, 391) consisted of three cattle or goats per test group with four test groups including an untreated control (Table 3). All treatments were applied at the start of the study with the exception of therapeutic products where the same group received different acaricides after a washout period during which the animals were not treated with any acaricide.

**Table 3:** Summary of the design of the *in vivo* studies.

Study number	Host species	Tick species	N =	Treatments by group [Active Ingredients, (Formulation/route) and administration day(s)]
**Exploratory studies**				
385	Cattle	R.m. W.A.	3	1: Negative Control
				2: Fipronil (pour-on) Day 0
				3: Cymiazole and Cypermethrin (dip and spray) Day 0 and every 7 days thereafter
				4: Ivermectin and Closantel (injection) Day 0
387	Cattle	R.m. E.A.	3	1: Negative Control
				2: Fipronil and Abamectin (pour-on) Day 0
				3: Cymiazole and Cypermethrin (dip and spray) Day 0 and every 7 days thereafter
				4: Fluazuron and Flumethrin (pour-on) Day 0
391	Goats	A.v.	3	1: Negative Control until day 60 then treated with Chlorfenvinphos and Alfamethrin (dip and spray) Day 60
				2: Fipronil (pour-on) Day 0
				3: Flumethrin (pour-on) Day 0
				4: Amitraz (Spray-dip) Day 0, Cymiazole and Cypermethrin (dip and spray) Day 18 and Flumethrin (dip and spray) on Day 32
389	Cattle	R.a.	3	1: Negative Control
				2: Fipronil and Abamectin (pour-on) Day 0
				3: Fipronil (pour-on) Day 0
				4: Flumethrin (dip and spray) Day 0, Amitraz (spray dip) Days 15 and 64, Cymiazole and Cypermethrin (dip and spray) (Day 29, Chlorfenvinphos and Alfamethrin (dip and spray) Day 57
**Confirmatory studies**				
386	Cattle	R.m. W.A.	6	1: Negative Control
				2: Fipronil (pour-on) Day 0
388	Cattle	R.m. E.A.	6	1: Negative Control
				2: Fipronil and Abamectin (pour-on) Day 0
392	Cattle	A.v.	6	1: Negative Control
				2: Flumethrin (dip and spray) Day 0
393	Goats	A.v.	6	1: Negative Control
				2: Flumethrin (pour-on) Day 0
390	Cattle	R.a.	6	1: Negative Control
				2: Fipronil and Abamectin (pour-on) Day 0

R.m. *Rhipicephalus microplus;* A.v. *Amblyomma variegatum;* R.a. *Rhipicephalus appendiculatus;* W.A: West Africa; E.A: East Africa; N = : number of animals per group.

##### 2.3.2.2 Confirmatory studies

Each of five confirmatory studies (study 386, 388, 390, 392, 393) consisted of six animals per test group with two groups, an untreated control and a second treated group treated with the most promising acaricide selected from the exploratory studies (Table 3). The number of animals per group and study design met international standards for Good Clinical Practice (VICH GL9). Goats, one of the preferred hosts of *A. variegatum,* along with cattle were used in these studies.

#### 2.3.3 Usage of tick stocks from the *in vitro* studies

The least susceptible stocks of each of the three tick species identified through the *in vitro* studies were used in *in vivo* efficacy studies to determine the most suitable commercial acaricides. For *R. microplus,* 3rd, 4th and 5th generation larvae of the least susceptible stock from each of East and West Africa were used whilst for the multi-host species *R*. *appendiculatus* and *A*. *variegatum,* 1st and 2nd generation adults from East African stocks were used.

#### 2.3.4 Tick exposure and efficacy assessment

A general outline of tick infestation is provided in this section with details of infestation days and count days by study shown in Supplementary Table 2. In general, acaricides were administered on Day 0 of the study to animals with patent tick infestations. Ticks were collected and counted based on their viability for a period immediately after the acaricide administration in order to assess the therapeutic tick kill efficacy. Additional tick infestations and counts were performed at various timepoints after acaricide administration to assess persistent efficacy, depending on the acaricide and formulation as advocated by the World Association for the Advancement of Veterinary Parasitology (WAAVP) (Holdsworth et al., 2006).

For *R. microplus*, whole body infestation was performed on cattle. Cattle were infested with ∼3000 larvae three times weekly for four weeks prior to acaricide administration for the purposes of randomisation and therapeutic efficacy assessments and ∼5000 larvae three times a week in weeks three, seven and eleven after acaricide administration to evaluate persistent efficacy. Larvae used in the artificial infestations were unfed and between two and 12 weeks old. The assessment criterion was the number of engorged female ticks dropping from the control and the acaricide treated groups on the various assessment days. Ticks were collected in the morning, washed, dried and counted.

For *A*. *variegatum*, animals were fitted with a jacket and infested with 20–30 unfed adult ticks of equal sex distribution under the jacket on the ventral side of the animal. Two infestations were performed prior to acaricide administration, one for the purposes of randomisation and a second immediately before acaricide administration for the purposes of therapeutic efficacy assessments. Persistent efficacy was assessed through fortnightly infestations after acaricide administration. *In situ* counts were performed at 48 and 72 h after infestation or acaricide administration followed by tick removal at 96 h. At each time point ticks were categorized as live or dead and free or attached. The reinfestation burden was standardized based on the number or ticks left attached. The assessment criterion was the number of live male and female ticks counted in the control and the acaricide treated groups on the various assessment days. After each tick count, three to five male ticks were left attached as an attraction stimulus for ticks during the following infestation.

A similar methodology to *A. variegatum* was used for *R*. *appendiculatus*, cattle were infested with 60 adult, unfed ticks of equal sex distribution (30 per ear), twice prior to acaricide administration (for randomisation and therapeutic efficacy assessments) and then once every two weeks for persistent efficacy assessments. Both ears were covered with ear bags immediately before the infestation and the required number of ticks was placed inside the bags that were subsequently closed. Tick counts and categorisation were performed as for *A. variegatum.*

#### 2.3.5 Selection and administration of test acaricides

Eight candidate acaricide products were selected for investigation in the exploratory studies (Table 3) and, of these, four went forward into the confirmatory studies (Table 3). Acaricide tested, doses, and routes of administration are shown in Table 2, Table 3

##### 2.3.5.1 Rhipicephalus microplus

Considering the extensive reported resistance of *R*. *microplus* in both East and West Africa, a range of pour-on formulations containing fipronil, flumethrin and the acarine growth regulator fluazuron, were assessed in exploratory studies. Spray-on formulations containing cymiazole and cypermethrin or amitraz were also assessed due to their significantly shorter wash-out period as well as an injectable formulation of ivermectin and closantel. All pour-on and injectable formulations were administered once on Day 0 whilst the spray-on was applied weekly.

Based on the efficacies in exploratory studies of 1% fipronil and the combination of 0.9% fipronil and abamectin, these products were selected for confirmatory studies.

##### 2.3.5.2 Amblyomma variegatum

An initial exploratory study using *A*. *variegatum* was performed in goats followed by confirmatory studies in goats and cattle.

Various pour-on and spray-on formulations were assessed in the initial exploratory study (Table 3). All the formulations were applied once-off directly to the groin and axillae, as recommended by the product label directions for targeting this species. In order to obtain additional data on repeat treatment, three different spray-on formulations were assessed in a single group. Amitraz was applied on Day 0, cymiazole and cypermethrin on Day 18 and flumethrin spray-on Day 32, once the persistent efficacy of the previous acaricide had worn off.

In two separate confirmatory studies, flumethrin pour-on was administered once to goats on Day 0 and the spray-on formulation was administered to cattle on Days 0, 25 and 35 in order to assess the impact of repeated applications on the persistent efficacy. Both formulations were administered directly to the axillae at the recommended dose of the manufacturer.

##### 2.3.5.3 Rhipicephalus appendiculatus

A combination of pour-on and spray-on formulations, together with different application routes, were assessed in the initial exploratory study. Fipronil 0.9% and abamectin 0.5% was administered as a stripe along the back (as recommended by the manufacturer) whilst 1% fipronil was administered as a single stripe to the ears using a paintbrush with a width of approximately 3 cm. All spray formulations were administered directly to the ears. These methods were chosen to target the predilection sites of the ticks. Due to the short wash out period of the four acaricidal spray/dip formulations, the therapeutic efficacy of these products was assessed in a single group through sequential administrations of separate acaricides after a resting period.

The combination of fipronil and abamectin were selected for further assessment in the confirmatory study. Additional *in situ* counts were performed such that the persistent efficacy up to 45 days at 72 h post infestation could be measured.

#### 2.3.6 Statistical analysis

##### 2.3.6.1 *In vitro* studies

All data was processed in MedCalc. A dose response analysis was performed with the probit regression function for each replicate and for all replicates of one stock added together. The log concentration with the corresponding probability was processed in Microsoft Excel and a graph was created. For each acaricide and each stock the log concentration (mg/mL) was plotted on the x-axis and the probability (0, 1-1,0) on the y-axis. The LC_50_ was determined for each strain.

The resistance ratio was calculated using the formula: LC_50_ resistant strain/LC_50_ susceptible strain.

##### 2.3.6.2 *In vivo* studies

Efficacy calculations were based on the WAAVP guidelines (Holdsworth et al., 2006) for evaluating the efficacy of acaricides against ticks (*Ixodidae*) on ruminants with adaptation for *R. microplus*. We defined therapeutic efficacy as the effect of an acaricide on pre-existing infestations (i.e. acaricide used to treat/control ticks) and persistent efficacy as the effect of an acaricide on infestations after application (i.e. acaricide used to prevent re-infestation). Arithmetic means were considered primary and efficacy was calculated as follows:

Efficacy (%) = 100 x (M_c_ – M_t_)/M_c_ where:

M_c_ = Mean number of live females detached engorged ticks for *R. microplus* in the control group. For *A. variegatum* and *R. appendiculatus:* mean number of live ticks on animals in the negative control group at each specific time point.

M_t_ = Mean number of live female detached engorged ticks for *R. microplus* in the treated group. For *A. variegatum* and *R. appendiculatus*: mean number of live ticks on animals in the acaricide treated group/s at each specific time point.

## 3 Results

### 3.1 *In vitro* studies - larval packet tests

**Fig. 1:**
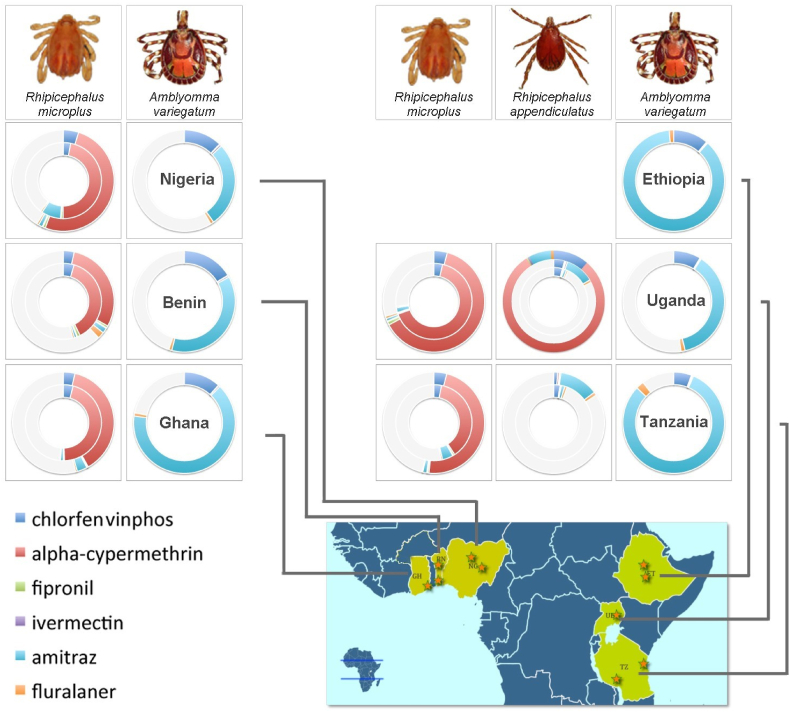
Relative, cumulative resistance factors of three tick species against six acaricides. Concentric circles within a single tick species and country represent individual tick stocks collected from different localities within each country (represented by stars on the map). The highest resistance factor (RF) was taken as reference (*A. variegatum* from Ethiopia) with the amount of incompletion in other circles representing the proportional reduction in RF. Detailed LC_50_ and RF values are provided in Table 4. The RF calculated for one Ugandan *R. appendiculatus* isolate (Serere 2) was capped in line with the reference as the value was considered an outlier and not representative.

To better understand how the individual tick stocks from each area compared to each other, the LC_50_s for each of the test acaricides were calculated for each stock. The added scores are presented in a doughnut chart to offer a visual and cumulative representation of the overall susceptibility of the different tick stocks (Fig. 1). A complete circle represents the highest cumulative LC_50_, and that of the other stocks represented by an incomplete circle, with the amount of incompletion representing the reduction in LC_50_ compared to the highest cumulative LC_50_ seen for *A. variegatum* in Ethiopia, used as the reference. The *R. appendiculatus* stock from Uganda was capped to equal the reference as the value was considered an outlier.

#### 3.1.1 Rhipicephalus microplus

##### 3.1.1.1 Screening assays

The LC_50_ for the different acaricides tested with the East and West African stocks is presented in Table 4. The highest LC_50_ for each of the acaricide classes is shown in bold. For East Africa, the Ugandan stocks showed the greatest number of highest LC_50_s (five out of six) with the Serere 1 stock being the least susceptible.

**Table 4:** Larval Packet Test results (LC_50_ (mg/mL) and resistance factor [RF]) of the selected tick stocks.

Stock	Chlorfenvinphos	Alpha-cypermethrin	Fipronil	Ivermectin	Amitraz	Furalaner	Cypermethrin
**L**C_**50**_**(mg/mL) of the different acaricides tested for the East African stocks of *Rhipicephalus microplus***							
Madibila (Tz)	1.18 [8.4]	0.72 [97.3]	0.14 [1.8]	0.62 [0.7]	**0.33 [11.8]**	0.06 [1.6]	
Chamakweza (Tz)	1.44 [10.3]	0.88 [118.9]	0.09 [1.1]	0.57 [0.6]	0.09 [3.2]	0.02 [0.5]	
Serere (Ug)	1.31 [9.4]	**1.23 [166.2]**	0.09 [1.1]	**0.64 [0.7]**	0.14 [5.0]	0.05 [1.4]	
Serere 1 (Ug)	**1.52 [10.9]**	1.18 [159.5]	**0.21 [2.6]**	0.53 [0.6]	0.06 [2.1]	**0.07 [1.9]**	
**L**C_**50**_**(mg/mL) of the different acaricides tested for the West African stocks of *Rhipicephalus microplus***							
Donga (Bn)	1.63 [11.6]	0.72 [97.3]	**0.25 [3.1]**	**0.69 [0.8]**	0.07 [2.5]	0.07 [1.9]	
Zou (Bn)	1.24 [8.9]	0.56 [75.7]	0.14 [1.8]	0.49 [0.6]	0.12 [4.3]	**0.20 [5.4]**	
Akusa (Gh)	1.46 [10.4]	0.84 [113.5]	0.05 [0.6]	0.64 [0.7]	0.08 [2.9]	0.03 [0.8]	
Narth Korkpe (Gh)	1.32 [9.4]	0.72 [97.3]	0.06 [0.8]	0.47 [0.5]	0.23 [8.2]	0.05 [1.4]	
Quanpam (Ng)	1.18 [8.4]	0.94 [127.0]	0.17 [2.1]	0.65 [0.7]	**0.62 [22.1]**	0.07 [1.9]	
**Soba (Ng)**	**1.74 [12.4]**	**0.96 [129.7]**	0.16 [2.0]	0.55 [0.6]	0.09 [3.2]	0.06 [1.6]	
**L**C_**50**_**(mg/mL) and resistance factor [RF] of the selected *Rhipicephalus microplus* tick stocks and the susceptible reference strain**							
Serere (Ug)	1.08 [7.7]	0.26 [35.1]	**0.075 [0.9]**	0.095 [0.1]	0.12 [4.3]	**0.051 [1.4]**	**0.815 [62.7]**
Donga (Bn)	**1.22 [8.7]**	**0.34 [45.9]**	0.067 [0.8]	**0.52 [0.6]**	**0.14 [5.0]**	0.031 [0.8]	0.036 [2.8]
Reference (SA)	0.14 [1.0]	0.0074 [1.0]	0.08 [1.0]	0.89 [1.0]	0.028 [1.0]	0.037 [1.0]	0.013 [1.0]
**L**C_**50**_**(mg/mL) and resistance factor [RF] of the *Amblyomma variegatum* tick stocks**							
Serere (Ug)	0.15 [22.7}	**0.021 [0.2]**	0.063 [0.2]	0.38 [0.8]	*0.84 [97.7]*	0.024 [3.4]	
Oromia (Et)	0.2 [30.3]	0.017 [0.1]	0.073 [0.2]	0.45 [1.0]	***2.02 [234.9]***	0.028 [3.9]	
Chamakweza (Tz)	0.097 [14.7]	0.019 [0.1]	**0.18 [0.6]**	0.47 [1.0]	*1.79 [208.1]*	**0.047 [6.6]**	
Akuse (Gh)	0.21 [31.8]	0.012 [0.1]	0.076 [0.3]	0.63 [1.6]	*1.51 [175.6]*	0.02 [2.7]	
Donga (Bn)	**0.31 [47.0]**	0.013 [0.1]	0.088 [0.3]	0.73 [1.6]	*0.91 [105.8]*	0.019 [2.7]	
Quanpam (Ng)	0.22 [33.3]	0.015 [0.1]	0.11 [0.3]	**0.85 [1.8]**	*0.64 [74.4]*	0.02 [2.8]	

Reference^a^ SA	0.0066 [1.0]	0.14 [1.0]	0.32 [1.0]	0.47 [1.0]	0.0086 [1.0]	0.0071 [1.0]	
**L**C_**50**_**(mg/mL) values and resistance factor (RF] for *Rhipicephalus appendiculatus* stock and the susceptible reference strain**							
Madibila (Tz)	0.043 [6.5]	0.033 [0.02]	0.098 [0.3]	0.38 [0.8]	0.03 [3.5]	0.018 [2.5]	
Chamakweza (Tz)	0.02 [3.0]	0.19 [1.4]	0.095 [0.3]	0.45 [1.0]	**0.26 [30.2]**	0.018 [2.5]	
Serere (Ug)	0.066 [10.0]	0.056 [0.4]	0.11 [0.3]	0.63 [1.3]	0.025 [2.9]	0.014 [2.0]	
Serere 1 (Ug)	0.069 [10.5]	0.046 [0.3]	0.34 [1.1]	0.73 [1.6]	0.24 [27.9]	0.018 [2.5]	
Serere 2 (Ug)	**0.22 [33.3]**	**64 [457.1]**	**0.38 [1.2]**	**0.85 [1.8]**	0.18 [20.9]	**0.019 [2.7]**	

Reference (SA)	0.0066 [1.0]	0.14 [1.0]	0.32 [1.0]	0.47 [1.0]	0.0086 [1.0]	0.0071 [1.0]	

Bold indicates stock with the highest LC_50_ for each compound.

Bn: Benin; Gh: Ghana; Ng: Nigeria; SA: Republic of South Africa; Tz: Tanzania; Ug: Uganda.

a As no reference strain was available for this tick species, the susceptible strain for *R*. *appendiculatus* was used as a reference. Further comment on the LD50 for Amitraz against *A variegatum* is provided in the body of the text.

From West Africa, both the Donga stock from Benin and the Soba stock from Nigeria showed the greatest number of highest LC_50_s (two out of six) however, it was the Donga and Quanpam isolates that showed the greatest resistance across all acaricide classes. Whilst either of these two stocks could be chosen, the Donga stock was selected as the least susceptible stock to take forward into *in vivo* studies.

##### 3.1.1.2 Resistance confirmation assays

The two least susceptible *R. microplus* stocks (Donga and Serere) were further compared to a susceptible strain from the Republic of South Africa to determine the level of resistance. The LC_50_ values and resistance ratios were presented (Table 4). The two tick stocks were found to show medium to high levels of resistance to chlorfenvinphos, alpha-cypermethrin and cypermethrin as well as amitraz.

#### 3.1.2 Amblyomma variegatum

The LC_50_ values are presented (Table 4).

In an attempt to estimate the resistance factor of these stocks to determine potentially effective acaricides, the level of resistance was estimated comparing the LC_50_ of the different *A*. *variegatum* stocks with the LC_50_ of another three-host tick, namely a susceptible strain of *R*. *appendiculatus*. The reason for selection of this species was because both species mainly feed as adults on cattle and may have alternative hosts for the immature life cycle stages. One-host ticks such as *R*. *microplus*, where all stages mainly feed on the same cattle and are therefore exposed to acaricide when an animal is treated, would be expected to develop resistance much faster than multi-host ticks. The outcome of this component of the study demonstrated a medium to high level of resistance for all tick stocks against chlorfenvinphos and amitraz (Table 4). The Ethiopian stock of *A*. *variegatum* (Oromia) was considered as the least susceptible one for East Africa while for West Africa all tick stocks showed a similar profile. The tick stock Donga from Benin was taken forward for *in vivo* evaluation.

#### 3.1.3 Rhipicephalus appendiculatus

*Rhipicephalus appendiculatus* was only isolated from East Africa. The LPT was performed for the two *R*. *appendiculatus* stocks (Madibila and Chamakwesa) originating from Tanzania, three stocks from Uganda (Serere, Serere 1 and Serere 2) and, as a susceptible control, a strain from the Republic of South Africa. The LC_50_ values and the resistance levels were shown (Table 4). The LC_50_ values for the two Tanzanian stocks were very similar for all acaricides except for amitraz where the Chamakweza stock showed a higher resistance factor (30). For the three Ugandan stocks, also very similar values were obtained except for the Serere 2 stock which showed high values for chlorfenvinphos (33) and especially for alpha-cypermethrin (457). The least susceptible was the stock Serere 2 and this was selected to take forward into *in vivo* studies.

### 3.2 *In vivo* studies – exploratory and confirmatory studies

The efficacy achieved against *R. microplus* in exploratory studies is summarised in Table 5 for the East African stock and in Table 6 for the West African stock. Detailed results are shown in Supplementary Table 3 and Supplementary Table 4. Whilst no products demonstrated a therapeutic efficacy >90% in the exploratory study, both 1% fipronil and the combination of 0.9% fipronil and abamectin showed a quicker onset of action in the confirmatory studies with no ticks observed from five days post administration onwards and a therapeutic efficacy exceeding 90%.

**Table 5:** *Comparative in vitro* (Larval Packet Test) and *in vivo* efficacy of commercial acaricides against an Ugandan stock of *Rhipicephalus microplus*.

Acaricides used in respective assessment			LPT	Exploratory Efficacy (%)			Confirmatory Efficacy (%)			
LPT	Exploratory	Confirmatory	LC50 (mg/mL)/[RF]	Therapeutic (average Day 0–21)	Persistent (average Day 42–56)	Persistent (Day 71–84)	Therapeutic (average Day 0–21)	Persistent 42 to 56	Persistent (average Day 42–56)	Persistent (average Day 98–112)
Alpha- cypermethrin			0.26 [35.1]							
Cypermethrin			0.815 [62.7]							
	Fluazuron and Flumethrin			60.8	94.7					
	Cymiazole and Cypermethrin			59.6	51.9					
Fipronil			0.075 [0.9]							
	Fipronil and Abamectin	Fipronil and Abamectin		87.5	91.3	76.4	93.6	ND	86.3	78.5

Cattle were infested with ticks weekly for 4 weeks prior to treatment and in weeks 3, 7 and 11 post treatment.

Group design and acaricide administration: Exploratory Study: 1: Negative Control, 2: Fipronil (pour-on) Day 0, 3: Cymiazole and Cypermethrin (dip and spray) Day 0 and every 7 days thereafter, 4: Ivermectin and Closantel (injection) Day 0.

Confirmatory Study: 1: Negative Control, 2: Fipronil (pour-on) Day 0.

**Table 6:** *Comparative in vitro* (Larval Packet Test) and *in vivo* efficacy of commercial acaricides against a Beninese stock of *Rhipicephalus microplus*.

Acaricides used in respective assessment			LPT	Exploratory Efficacy (%)			Confirmatory Efficacy (%)		
LPT	Exploratory	Confirmatory	LC50 (mg/mL)/[RF]	Therapeutic (average Day 0 to Day 21)	Persistent (average Day 42–56)	Persistent (Day 71–84)	Therapeutic (average Day 0 to Day 21)	Persistent (average Day 42–56)	Persistent (Day 70–84)
Cypermethrin			0.036 [2.8]						
	Cymiazole and Cypermethrin			49.7	75.1				
Fipronil	Fipronil	Fipronil	0.067 [0.8]	84.6	100	94.8	91	84.3	51.9
Ivermectin			0.52 [0.6]						
	Ivermectin and Closantel			74.5	16.4				

Cattle were infested with ticks weekly for 4 weeks prior to treatment and in weeks 3, 7 and 11 post treatment.

Group design and acaricide administration: Exploratory Study: 1: Negative Control, 2: Fipronil and Abamectin (pour-on) Day 0, 3: Cymiazole and Cypermethrin (dip and spray) Day 0 and every 7 days thereafter, 4: Fluazuron and Flumethrin (pour-on) Day 0.

Confirmatory Study: 1: Negative Control, 2: Fipronil and Abamectin (pour-on) Day 0.

Persistent efficacy, assessed in the same confirmatory studies, demonstrated the combination therapy of fipronil and abamectin controlled >85% of ticks for up to seven weeks post treatment whereas fipronil alone achieved the same level of control for only three weeks in East African and West African strains.

Against *A. variegatum,* although onset of action was slow, taking 96 h for the control of existing tick infestations, flumethrin pour-on controlled >90% of ticks on goats within 48 h post tick infestation up to 28 Days post treatment (summarised in Table 7 and detailed in Supplementary Table 5). Fipronil was not effective in controlling this tick species.

**Table 7:** Comparative *in vitro* (Larval Packet Test) and *in vivo* efficacy of commercial acaricides against a Beninese stock of *Amblyomma variegatum*.

Acaricides used in respective assessment			LPT	Exploratory Efficacy^d^ (%)			Confirmatory Efficacy^d^ (%)				
LPT	Exploratory	Confirmatory	LC50 (mg/mL)/[RF]	Therapeutic	Persistent (2 weeks)	Persistent (4 weeks)	Therapeutic	Persistent (2 weeks)	Persistent (4 weeks)	Persistent (6 weeks)	Persistent (8 weeks)
Chlorfenvinphos			0.31 [47.0]								
	Chlorfenvinphos and Alfamethrin^a^			57.9							
Alpha- cypermethrin	Flumethrin pour-on^a^	Flumethrin pour-on^b^	0.013 [0.1]	23.3	94.5	81.0	33.7	85.3	82.0	73.9	59.2
	Flumethrin dip and spray^a^			100	91.4						
		Flumethrin dip and spray^c^					99.0	38.0			
	Cymiazole and Cypermethrin^a^			100	60.3						
Fipronil			0.088 [0.3]								
	Fipronil and Abamectin^a^			36.7	29.1	13.8					
Amitraz	Amitraz^a^		0.91 [105.8]	100	34.5						

Group design and acaricide administration.

a Exploratory Study (Goats)1: Negative Control until Day 60 then treated with Chlorfenvinphos and Alfamethrin (dip and spray) Day 60, 2: Fipronil (pour-on) Day 0, 3: Flumethrin (pour-on) Day 0. 4: Amitraz (Spray-dip) Day 0, Cymiazole and Cypermethrin (dip and spray) Day 18 and Flumethrin (dip and spray) on Day 32.

b Confirmatory study (Goats): 1: Negative Control, 2: Flumethrin (pour-on) Day 0.

c Confirmatory study (Cattle): 1: Negative Control 2: Flumethrin (dip and spray) Days 0, 25 and 35.

d Efficacy reported at 48 h post acaricide administration (therapeutic) or challenge (Persistent).

Results for the various spray-on formulations against *A. variegatum* (Supplementary Table 6) are presented as Days post acaricide administration for comparison purposes. Amitraz, cymiazole and cypermethrin and flumethrin spray-on all demonstrated a quick onset of action in goats, controlling 100% of ticks within 48 h after treatment. Persistent activity was observed for flumethrin dip and spray in goats with >90% control of ticks within 48 hat two weeks post treatment.

In cattle the spray-on formulation of flumethrin had a similarly rapid onset of action as seen in goats with a therapeutic efficacy of 99%, however no persistent efficacy was observed (Summarised in Table 7 and detailed in Supplementary Table 7).

Against *R. appendiculatus* both fipronil based pour-on formulations, 0.9% fipronil and 0.5% abamectin and 1% fipronil controlled ≥90% of ticks within 48 h after acaricide administration (Summarised in Table 8 and detailed in Supplementary Table 8). Whilst applying the dose of fipronil as a single stripe directly to the ears resulted in a quicker onset of action, application of a larger dose (fipronil and abamectin) as a stripe along the back resulted in a longer persistent efficacy killing >90% within 48 h of infestation up to four weeks post acaricide administration. The efficacy of the combination product was further evaluated as a stripe along the backline in a confirmatory study in which the onset of action was slower, taking 72 h to kill >90% of the existing tick infestation. Furthermore, persistent efficacy at 4 weeks post treatment was also reduced, taking 72 h to kill >90% of the newly infested ticks (Table 8 and Supplementary Table 10).

**Table 8:** *Comparative in vitro* (Larval Packet Test) and *in vivo* efficacy of commercial acaricides against an Ugandan stock of *Rhipicephalus appendiculatus* East Africa.

Acaricides used in respective assessment			LPT	Pilot Exploratory Efficacy^a^ (%)				Confirmatory Efficacy^a^ (%)			
LPT	Exploratory	Confirmatory	LC50 (mg/mL)/[RF]	Therapeutic	Persistent (2 weeks)	Persistent (4 weeks)	Persistent (8 weeks)	Therapeutic	Persistent (2 weeks)	Persistent (4 weeks)	Persistent (6 weeks)
Chlorfenvinphos			0.22 [33.3]								
	Chlorfenvinphos and Alfamethrin			33.3							
Alpha- cypermethrin	Flumethrin dip and spray		64 [457.1]	100							
	Cymiazole and Cypermethrin			91.7							
Fipronil	Fipronil (pour-on)		0.38 [1.2]	100	95.1	75.2	54.9				
	Fipronil and Abamectin	Fipronil and Abamectin		99.4	97.6	95.4	11.8	42.4	92.9	68.4	46.6
Amitraz	Amitraz		0.18 [20.9]	100							

Group design and acaricide administration.

Exploratory Study: 1: Negative Control, 2: Fipronil and Abamectin (pour-on) Day 0 backline, 3: Fipronil (pour-on) Day 0 painted on to ears, 4: Flumethrin (dip and spray) Day 0, Amitraz (spray dip) Days 15 and 64, Cymiazole and Cypermethrin (dip and spray) Day 29, Chlorfenvinphos and Alfamethrin (dip and spray) Day 57 – all applied directly to the ears.Confirmatory study: 1: Negative Control, 2: Fipronil and Abamectin (pour-on) Day 0.

a Efficacy reported at 48 h post acaricide administration (therapeutic) or challenge (persistent).

Of the spray-on formulations, flumethrin, amitraz, and cymiazole and cypermethrin controlled >90% of ticks within 24 h after application (Table 8 and Supplementary Table 9).

All cattle and goats were normal throughout the studies and no adverse events were observed.

## 4 Discussion

Most comparable studies examining acaricide resistance in this region have focused on *R. microplus* and specifically on organophosphate and synthetic pyrethroid (SP) compounds, whilst this study extended the assessment to include resistance to the newer compounds ivermectin, fipronil, fluralaner, and combination acaricides, in three of the most important tick species in Africa. The objective of this series of studies, as opposed to a broad screening exercise, was to provide a deeper evaluation of acaricides by comparing *in vitro* bioassays with *in vivo* efficacy and to provide an indicator of the sustainability of available acaricide classes within current control strategies. To reflect the profile of the products used in the field, some combination acaricides were tested *in vivo*, to evaluate the potential of associating several compounds with different Mode of Action to control resistant populations.

The LPT results for all three tick species demonstrated medium to high levels of resistance to chlorfenvinphos and amitraz. *Rhipicephalus microplus* also demonstrated high levels of resistance to SPs. *Rhipicephalus appendiculatus*, in two stocks from Uganda also demonstrated medium levels of resistance to ivermectin. Within this project, most strains of *R. appendiculatus* were susceptible to the SP alpha-cypermethrin, with the reason for one extremely high value for alpha-cypermethrin in the third Ugandan stock being unclear. Overall, within the *in vitro* tests, the products for which no resistance was detected contained fipronil and to a lesser extent ivermectin. This may relate to the relative lack of usage of these two chemicals as livestock acaricides in this region, the former due to its long withdrawal period and the latter due to cost and lack of efficacy in controlling tsetse flies (Githaka et al., 2022). Whilst the applied LPT methodology, based on the 2004 FAO guidelines, is oriented towards one-host ticks, validation of results for multi-host ticks was based on obtaining a full dose response curve, thus permitting the calculation of representative LC50 values, similar to previously reported approaches (Chitombo et al., 2021; Makuvadze et al., 2020). Furthermore, although isoxazolines are known to act primarily systemically, a dose response to topical exposure with fluralaner was observed, providing an indication of susceptibility to this class.

To the best of the authors knowledge, there are currently no registered isoxazoline based formulations for use in production animals in Africa however, fluralaner has recently been registered for cattle in Brazil and was included to provide a reference point for the current status of susceptibility of field isolates to this class of acaricide. The *R. appendiculatus* stocks demonstrated a moderately higher level of tolerance to fluralaner in comparison to the reference isolate. In the absence of isoxazoline products in Africa, and therefore no selection pressure, this is unlikely to be of concern but is an important finding for future acaricide selection.

The results reported here extend previous research findings using similar ticks extracted from the field in western and central Uganda (Vudriko et al., 2016); Benin (Adehan et al., 2016) and Tanzania (Nagagi et al., 2020). Whilst the methodology for determining the resistance status in these studies was not identical, the trend towards resistance was similar. These researchers utilised ticks collected in 2013–2015, 2013, and 2017–2019 respectively. Our research utilised ticks collected in 2016–2017. These combined findings offer up further clarity on the establishment and spread of resistance in defined strains of identified tick species, to several classes of acaricides in commercial usage in Africa.

This study took the least susceptible strains *in vitro* into *in vivo* studies, making this study more extensive than those previously reported and permitted examination of, for example, the efficacy of combination products and the effect of administration route on the onset and duration of efficacy. Whilst guidance on clinical trial design as well as the determination of efficacy of acaricides against one host ticks is well documented, there are limited detailed resources for multi-host ticks, particularly for *A. variegatum.* The *in vivo* studies were therefore designed to account for host predilection sites and attachment stimuli whilst evaluation of acaricidal efficacy was performed at multiple timepoints after acaricide administration or tick challenge. Route of application and dose rate for pour-on products was found to be an important consideration, for example, against *R. appendiculatus,* direct application to the ears resulted in more rapid activity whereas a stripe along the back resulted in improved persistance. Thus, whilst considerably more time consuming and expensive than *in vitro* tests alone, the *in vivo* studies provide considerably more insight.

Whilst *in vitro*, ivermectin was effective against *R. microplus, in vivo*, when administered as an injectable formulation it was not effective. The mode of action of ivermectin, acting systemically, may have contributed to the differences in efficacy, demonstrating the importance of validating results using additional alternative *in vitro* models such as the adult immersion test or through *in vivo* assays.

With reports of multiple resistance against several acaricide classes becoming increasingly prevalent (Githaka et al., 2022) we aimed to evaluate resistance to formulations containing single acaricide classes *in vitro* and contrast these with combination acaricides assessed *in vivo.* Reports have demonstrated the synergistic behaviour of combinations of SPs and amidines in which the addition of permethrin to amitraz increased the acaricidal efficacy of amitraz alone, by up 54 times in an LPT (Li et al., 2007). In contrast, the addition of cymiazole (an amidine) to cypermethrin provided limited increased therapeutic control in our target animal studies. Following a different approach, the addition of the acarine growth regulator, fluazuron, did improve persistent efficacy.

Fipronil was effective against both West and East African *R. microplus* isolates although efficacy after six weeks dropped sharply.

In contrast to *in vitro* assays where severe resistance of all *A. variegatum* stocks to amitraz was observed, therapeutic control was possible through the administration of SPs or amitraz in spray formulations directly to the predilection sites in both cattle and goats, however with limited persistent efficacy. This stark contrast suggests interpreting the *in vitro* data in the context of the target animal results. Resistance factors for *A. variegatum* were calculated using a proxy susceptible reference tick species, *R*. *appendiculatus,* which may have overestimated the resistance to amitraz. Organophosphates (OPs) however, were not effective against *A*. *variegatum* or *R. appendiculatus,* either as a mono-formulation *in vitro*, or in combination with the SP alfamethrin, when assessed *in vivo.* Although there is limited data on the resistance of *Amblyomma* spp., reports from Zambia (Muyobela et al., 2015) on *A*. *variegatum* and from Zimbabwe (Makuvadze et al., 2020) on *A. hebraeum* presented LC_50_ values ranging from 0.009 to 0.02 %, considerably lower than the range of 0.8–2% as found in our LPT results. Makuvadze et al. (2020) also noted that their *R. appendiculatus* reference isolate was three times more sensitive than a reference *A. hebraeum.* The differences in observed tick tolerance to acaricides between *in vitro* assays and assessments in the target animals further reflect the challenges of interpreting *in vitro* data into *in vivo* protection. Pour-on products containing fipronil offered residual control for four weeks (with slow onset of action) against *R. appendiculatus* but were not effective against *A. variegatum,* even when applied directly to the predilection sites.

Flumethrin pour-on was the only product to provide persistent efficacy up to one month control at 72 h post treatment), against *A. variegatum*, following product application directly to tick predilection sites on the host. Flumethrin is recognized as providing superior acaricidal activity compared to other second generation synthetic pyrethroids due to the addition of the fluorine molecule into the structure (Khambay and Jewess, 2004). The observed differences in the efficacy of spray and pour-on formulations of flumethrin are likely explained by the dose and formulation the animals were exposed to, rather than the host species differences. Goats treated with the pour-on received a dose of 10 mg/kg whilst spray formulations were administered to cattle at a lower dose and only at tick attachment sites.

In addition to generating additional data on the susceptibility of these tick stocks to chemical classes currently used to treat livestock ticks in Africa, novel data on the response of African isolates to isoxazolines was also generated. The results demonstrated moderate levels of tolerance to this class, with higher RFs in multi-host ticks. This may represent normal biological variation in the response of different isolates and provides a baseline for interpreting responses to isoxazolines in future work.

The results from this work define the acaricide resistance status of economically important ticks of livestock from Sub-Saharan Africa. Whilst the scope of the results is limited to six countries and up to three stocks per country, they confirm the value and limitations of commercially available acaricides in Africa and the importance of responsible acaricide use in limiting the further development of resistance. It is notable that there is a lack of acaricidal products available for use in lactating animals. With this understanding on the in-field situation of tick resistance and acaricidal management options, it is clear that future Research & Development (R&D) priorities need to include alternative acaricides (be they synthetically derived or acquired from native botanical extracts), novel administration strategies for these compounds, integrated tick management strategies as well as more investment in vaccinology targeting ruminants for ticks. As ticks can play a primary role in vector borne disease transmission additional R&D foci on in-field/pen-side diagnostic tests targeting vector borne diseases of ruminants is also crucial to support a broader set of management tools in animal health and productivity.

## 5 Conclusion

There was some discordance between efficacy indicated by LPT and *in vivo* results. This observation calls for more research into accurate and affordable assessment methods for acaricide resistance.

No single active or product was effective against all three tick species, emphasising the need for the development of alternative tick management solutions.

Future research emphasis needs to equally focus on identifying and developing new classes of acaricide compounds as well as prioritising research in vaccinology targeting single and multi-host ticks of ruminants.

## Animal welfare statement

Animal experimentation undertaken within this research project met the requirements of the International Guiding Principles for Biomedical Research Involving Animals as issued by the Council for the International Organizations of Medical Sciences.

## Funding

This work forms part of a 10.13039/100000865Bill & Melinda Gates Foundation (10.13039/100000865BMGF) supported project, aiming to improve productivity, profitability, and sustainability of resource-poor farming systems in East and West Africa by evaluating existing and establishing new ecto- and endo-parasite control measures for livestock. This work was supported, in whole or in part, by the 10.13039/100000865BMGF [Grant Number OPP1155997]. Under the grant conditions of the Foundation, a Creative Commons Attribution 4.0 Generic License has already been assigned to the Author Accepted Manuscript version that might arise from this submission.

## CRediT authorship contribution statement

**Alec Evans:** Investigation, Project administration, Writing – review & editing. **Maxime Madder:** Supervision, Visualization, Writing – review & editing. **Josephus Fourie:** Conceptualization, Methodology. **Lénaïg Halos:** Conceptualization. **Bersissa Kumsa:** Investigation, Writing – review & editing. **Elikira Kimbita:** Investigation, Writing – review & editing. **Joseph Byaruhanga:** Investigation, Writing – review & editing. **Frank Norbert Mwiine:** Investigation, Writing – review & editing. **Dennis Muhanguzi:** Investigation, Writing – review & editing. **Safiou Bienvenu Adehan:** Investigation, Writing – review & editing. **Alassane Toure:** Investigation, Writing – review & editing. **Jahashi Nzalawahe:** Investigation, Writing – review & editing. **Fred Aboagye-Antwi:** Investigation, Writing – review & editing. **Ndudim Isaac Ogo:** Investigation, Writing – review & editing. **Leon Meyer:** Investigation, Visualization, Writing – review & editing. **Frans Jongejan:** Investigation. **Imad Bouzaidi Cheikhi:** Investigation. **Maggie Fisher:** Visualization, Writing – original draft, Writing – review & editing. **Peter Holdsworth:** Visualization, Writing – original draft, Writing – review & editing.

## Declaration of competing interest

The authors declare that they have no competing interest.
