# Supplementary material for: Acaricide resistance status of livestock ticks from East and West Africa and in vivo efficacy of acaricides to control them
Source: Int J Parasitol Drugs Drug Resist. 2024 May 6;25:100541. doi: 10.1016/j.ijpddr.2024.100541 (PMC11133915; doi:10.1016/j.ijpddr.2024.100541)
Supplement: Multimedia component 1 [file mmc1.docx]

Supplementary tables – final

Supplementary Table 1. Locality and countries from which stocks of *Rhipicephalus microplus*, *Amblyomma variegatum* and *Rhipicephalus appendiculatus* were collected

Supplementary Table 2. Summary of tick infestations and tick counts

Supplementary Table 3. Efficacy of fipronil, fipronil and abamectin, flumethrin and fluazuron, ivermectin and closantel and cymiazole and cypermethrin against *Rhipicephalus microplus* (West and East African isolates) in the exploratory studies (nos. 385 & 387)

Supplementary Table 4. Efficacy of fipronil, fipronil and abamectin, against *Rhipicephalus microplus* (West and East African isolates) in the confirmatory studies (nos. 386 & 388)

Supplementary Table 5. Therapeutic and persistent efficacy of fipronil and abamectin and flumethrin pour-on against *Amblyomma variegatum* in the exploratory study on goats (no. 391)

Supplementary Table 6. Therapeutic and persistent efficacy of spray-on formulations; amitraz, cymiazole and cypermethrin, flumethrin and chlorfenvinphos and alfamethrin against *Amblyomma variegatum* in the exploratory study on goats (no. 391)

Supplementary Table 7. Therapeutic and persistent efficacy of 1% flumethrin pour-on on goats and 2% flumethrin spray-on on cattle against *Amblyomma variegatum* in the confirmatory studies (nos. 392 & 393)

Supplementary Table 8. Therapeutic and persistent efficacy of pour-on formulations; 0.9% fipronil and 0.5% abamectin and 1% fipronil against *Rhipicephalus appendiculatus* ticks in the exploratory study (no. 389)

Supplementary Table 9. Therapeutic efficacy of spray-on formulations; flumethrin dip and spray, amitraz, cymiazole and cypermethrin and chlorfenvinphos and alfamethrin against *Rhipicephalus appendiculatus* ticks in the exploratory study (no. 389)

Supplementary Table 10. Therapeutic and persistent efficacy of fipronil and abamectin against *Rhipicephalus appendiculatus* ticks in the confirmatory study (no. 390)

--------------------------------------------------------------------------------------------------------------------------------------

**Supplementary Table 1. Locality and countries from which stocks of *Rhipicephalus microplus*, *Amblyomma variegatum* and *Rhipicephalus appendiculatus* were collected**

|  | **Locality, Country** |  | ***R. microplus*** | ***A. variegatum*** | ***R. appendiculatus*** |
| --- | --- | --- | --- | --- | --- |
| **East Africa** |  |  |  |  |  |
|  | Madibila, Tanzania |  | x | x | x |
|  | Chamakweza, Tanzania |  | x | x | x |
|  | Serere, Uganda |  | x | x | x |
|  | Serere 1, Uganda |  | x |  | x |
|  | Serere 2, Uganda |  |  |  | x |
|  | Oromia, Ethiopia |  |  | x |  |
|  |  |  |  |  |  |
| **West Africa** |  |  |  |  |  |
|  | Donga, Benin |  | x | x |  |
|  | Zou, Benin |  | x |  |  |
|  | Akuse, Ghana |  | x | x |  |
|  | Narth Korkpe, Ghana |  | x |  |  |
|  | Quanpam, Nigeria |  | x | x |  |
|  | Soba, Nigeria |  | x |  |  |

**Supplementary Table 2. Summary of tick infestations and tick counts**

| **Study no.** | **(No. ticks) and infestation days** | **Assessment days** |
| --- | --- | --- |
| **Exploratory studies** | | |
| 385 | Cattle were infested with approximately3 000 *R*. *microplus* larvae per bovine on Days -28, -25, -23, -21, ‑18, -15, -14, -10, -8 and -4 for the purposes of randomisation and therapeutic efficacy assessments and approximately 5 000 *R*. *microplus* larvae) Days 21, 25, 28, 49, 52, 56, 77, 80 and 84 for persistent efficacy assessments. | Therapeutic efficacy: fully engorged female ticks collected and counted from Days 1 to 21.  Persistent efficacy: engorged female ticks were collected and counted from Days 42 to 56 (all groups) Days 71 to 84, Days 99 to 112 (groups 1 to 3). |
| 387 | Cattle were infested with approximately 3 000 *R*. *microplus* larvae applied per bovine on Days -28, -25, -23, -20, ‑18, -15, -13, -11 -8 and -4 for the purposes of randomisation and therapeutic efficacy assessments and approximately 5 000 *R*. *microplus* larvae) Days 21, 24, 28, 49, 52, 56, 77 and 80 for persistent efficacy assessments. | Therapeutic efficacy: fully engorged female ticks were collected and counted from Days 1 to 21.  Persistent efficacy: fully engorged female ticks were collected and counted from Days 42 to 56 for all groups and Days 70 to 83 for groups 2 and 4. |
| 391 | Approximately 30 adult unfed *A*. *variegatum* ticks (±15 males and ±15 females) were applied per goat on Day ‑14 for randomisation purposes and Day ‑4 for evaluation of therapeutic efficacy. Goats were reinfested (approximately 20 adult unfed *A*. *variegatum* ticks (±10 males and ±10 females)) on Days 14 and 28 to assess persistent efficacy in groups 2 and 3 and therapeutic efficacy in group 4. Group 3 were also infested on Day 42 to assess persistent efficacy, Group 4 were infested on Days 42 and 56 to assess therapeutic efficacy | Tick counts were performed at 48, 72 and 96 h (±4 h) after infestation or treatment administration. |
| 389 | 30 adult, unfed *R*. *appendiculatus* ticks per ear) applied per bovine on Day ‑6 for randomisation purposes, on Day -1 for evaluation of therapeutic efficacy, Days 14, 28, 56 and 63 to assess persistent efficacy for groups 2 and 3, and therapeutic efficacy for the respective treatments in group 4. | In-situ tick counts were conducted on Day 1 (groups 1 to 3) and Day 65 (all groups). Ticks were counted and removed on Days -4, 2, 16, 30, 58 (48 h ± 4 h after tick infestation or treatment administration for groups 2 and 3, or 24 h ± 4 h after treatment administration for group 4) and 66 (72 h ± 4 h after tick infestation). |
|  | | |
| **Confirmatory studies** | | |
| 386 | Cattle were infested with approximately 3 000 *R*. *microplus* larvae per bovine on Days ‑27, ‑25, ‑22, ‑20, ‑18, ‑15, ‑13, ‑11, ‑8, ‑4 and ‑1 for the purposes of randomisation and therapeutic efficacy assessments and approximately 5 000 *R*.*microplus* larvae on Days 21, 24, 28, 49, 52, 55, 77 and 80 for persistent efficacy assessments. | Fully engorged female ticks were collected and counted from Days 1 to 21 to assess therapeutic efficacy. Fully engorged female ticks were collected and counted from Days 42 to 56 and Days 70 to 83, to assess persistent efficacy. |
| 388 | Cattle were infested with approximately 3 000 *R*. *microplus* larvae per bovine on Days -27, -25, -22, -20, ‑18, -15, -13, -11, -8, -4 and -1 for the purposes of randomisation and therapeutic efficacy assessments and approximately 5 000 *R*. *microplus* larvae on Days 21, 25, 28, 49, 53, 56, 77, 80 and 84 for testing persistent efficacy. | Fully engorged female ticks were collected and counted from Days 1 to 21 to assess therapeutic efficacy, and from Days 42 to 56, Days 70 to 84 and Days 98 to 112, to assess persistent efficacy. |
| 392 | Cattle were infested with approximately 30 adult unfed *A*. *variegatum* ticks (±15 males and ±15 females) on Day -6 for randomisation purposes, Day -1 for therapeutic efficacy and on Days 14, 28 and 42 to assess persistent efficacy. During tick removal, up to five male ticks were left attached to the animal. The exact number of remaining male ticks left on the animal were recorded. Prior to the subsequent tick infestation, an i*n situ* count was performed to confirm the number of attached male ticks and this number was subtracted from the number of male ticks being infested on that day. Additional, in situ tick counts were performed at 48 h (± 4 h) and 72 h (± 4 h) after the acaricide administration or tick infestation. | Tick counts were performed at 48, 72 and 96 h (±4 h) after infestation or treatment administration. |
| 393 | On Day -14, all goats were fitted with a corset and artificially infested with 20 (± 2) adult, unfed *A*. *variegatum* ticks (10 males and 10 females). Approximately 20 (± 2) adult, unfed *A*. *variegatum* ticks of equal sex distribution were infested for immediate therapeutic efficacy on Day -2 and for persistent efficacy on Days 14, 28, 42 and 56. | Additional in situ tick counts were performed at 48 (± 4 h) and 72 (± 4 h) hours after the acaricide administration or tick infestation. Tick counts and removal were performed at 96 (± 4 h) h after the acaricide administration or infestation. |
| 390 | 30 (even sex distribution) adult, unfed *R*. *appendiculatus* ticks per ear) applied per cow on Day ‑6 for randomisation purposes, on Day -1 for evaluation of therapeutic efficacy, and on Days 14, 28 and 42 to assess persistent efficacy. | Ticks were removed and counted on Day ‑3 (72 h ± 4 h after infestation) for randomisation and ranking purposes. Ticks were counted in situ on Day 2 (48 h ± 4 h after acaricide treatment) and on Days 16, 30 and 44 (48 h ± 4 h after infestation) and removed on Day 3 (72 h ± 4 h after acaricide treatment) and on Days 17, 31 and 45 (72 h ± 4 h after infestation). |

**Supplementary Table 3. Efficacy of fipronil, fipronil and abamectin , flumethrin and fluazuron, ivermectin and closantel and cymiazole and cypermethrin against *Rhipicephalus microplus* (West and East African isolates) in the exploratory studies (nos. 385 & 387)**

|  | **West Africa** | | | | | | **East Africa** | | | | | |
| --- | --- | --- | --- | --- | --- | --- | --- | --- | --- | --- | --- | --- |
| Day | **Fipronil** | | **Cymiazole and cypermethrin** | | **Ivermectin and closantel** | | **Fipronil and abamectin** | | **Cymiazole and cypermethrin** | | **Fluazuron and flumethrin** | |
|  | Mean | Effic. | Mean | Effic. | Mean | Effic. | Mean | Effic. | Mean | Effic. | Mean | Effic. |
| 0 - 21 | 169.3 | 84.6 | 553.0 | 49.7 | 280.0 | 74.5 | 138.3 | 87.5 | 447.0 | 59.6 | 433.7 | 60.8 |
| 42 - 56 | 0.0 | 100 | 85.7 | 75.1 | 288.0 | 16.4 | 38.7 | 91.3 | 212.7 | 51.9 | 23.3 | 94.7 |
| 71 - 84 | 9.0 | 94.8 | 10.0 | 94.2 |  |  | 46.0 | 76.4 |  |  | 100.7 | 48.3 |
| 99 - 112 | 30.0 | 32.8 | 10.7 | 76.1 |  |  |  |  |  |  |  |  |

Mean: Mean tick count; Effic.: Percent efficacy compared to untreated control

**Supplementary Table 4. Efficacy of fipronil, fipronil and abamectin, against *Rhipicephalus microplus* (West and East African isolates) in the confirmatory studies (nos. 386 & 388)**

|  | **West Africa** | | **East Africa** | |
| --- | --- | --- | --- | --- |
|  | **Fipronil** | | **Fipronil and abamectin** | |
| Day | Mean | Efficacy | Mean | Efficacy |
| 0 to 21 | 4.0 | 91.0 | 21.7 | 93.6 |
| 42 to 56 | 40.0 | 84.3 | 0.0 | -* |
| 70 to 84 | 125.3 | 51.9 | 59.2 | 86.3 |
| 98 to 112 | - | - | 86.5 | 78.5 |

** insufficient ticks collected from the control group during this period*

Mean: Mean tick count; Efficacy: Percent efficacy compared to untreated control

**Supplementary Table 5. Therapeutic and persistent efficacy of fipronil and abamectin and flumethrin pour-on against *Amblyomma variegatum* in the exploratory study on goats (no.391)**

| **Day** |  | **Control** | **Fipronil** | | **Flumethrin pour-on** | |
| --- | --- | --- | --- | --- | --- | --- |
|  | Timepoint | Mean | Mean | Efficacy | Mean | Efficacy |
| 2 | 48 h post acaricide | 20.0 | 12.7 | 36.7 | 15.3 | 23.3 |
| 3 | 72 h post acaricide | 20.3 | 8.3 | 59.0 | 9.0 | 55.7 |
| 4 | 96 h post acaricide | 19.0 | 7.0 | 63.2 | 0.3 | 98.2 |
| 16 | 48 h post infestation | 18.3 | 13.0 | 29.1 | 1.0 | 94.5 |
| 17 | 72 h post infestation | 18.7 | 10.7 | 42.9 | 1.0 | 94.6 |
| 18 | 96 h post infestation | 18.3 | 9.0 | 50.9 | 1.0 | 94.5 |
| 30 | 48 h post infestation | 19.3 | 16.7 | 13.8 | 3.7 | 81.0 |
| 31 | 72 h post infestation | 19.3 | 13.7 | 29.3 | 1.7 | 91.4 |
| 32 | 96 h post infestation | 19.3 | 13.7 | 29.3 | 1.3 | 93.1 |
| 44 | 48 h post infestation | 19.3 | - | - | 6.9 | 63.8 |
| 45 | 72 h post infestation | 19.7 | - | - | 7.0 | 64.4 |
| 46 | 96 h post infestation | 19.7 | - | - | 7.0 | 64.4 |

Mean: Mean tick count; Efficacy: Percent efficacy compared to untreated control

**Supplementary Table 6. Therapeutic and persistent efficacy of spray-on formulations; amitraz, cymiazole and cypermethrin, flumethrin and chlorfenvinphos and alfamethrin against *Amblyomma variegatum* in the exploratory study on goats (no.391)**

|  | **Amitraz** | | **Cymiazole and cypermethrin** | | **Flumethrin spray-on** | | **Chlorfenvinphos and alfamethrin** | |
| --- | --- | --- | --- | --- | --- | --- | --- | --- |
| **Days post acaricide*** | **Control mean (Mean)** | **Efficacy** | **Control mean (Mean)** | **Efficacy** | **Control mean (Mean)** | **Efficacy** | **Pre-count mean (post-count-mean)** | **Efficacy** |
| **Therapeutic efficacies** | | | | | | | | |
| 2 | 20.0 (0.0) | 100 | 18.0 (0.0) | 100 | 19.3 (0.0) | 100 | 19.0 (8.0) | 57.9 |
| 3 | 20.3 (0.0) | 100 | 18.0 (0.0) | 100 | 19.0 (0.0) | 100 | 19.0 (2.3) | 87.7 |
| 4 | 19.0 (0.0) | 100 | 18.0 (0.0) | 100 | 19.0 (0.0) | 100 | 19.0 (1.0) | 94.7 |
| **Persistent efficacies** | | | | | | | | |
| 10 | - | - | - | - | - | - | - | - |
| 12 | - | - | 19.3 (7.7) | 60.3 | 19.3 (1.7) | 91.4 | - | - |
| 13 | - | - | 19.3 (7.3) | 62.1 | 19.7 (1.3) | 93.2 | - | - |
| 14 | - | - | 19.3 (4.7) | 75.9 | 19.7 (0.7) | 96.6 | - | - |
| 16 | 18.3 (12.0) | 34.5 | - | - | - | - | - | - |
| 17 | 18.7 (8.3) | 55.4 | - | - | - | - | - | - |
| 18 | 18.3 (8.0) | 56.4 | - | - | - | - | - | - |
| 24 | - | - | - | - |  |  | - | - |
| 26 | - | - | - | - | 19.0 (6.7) | 64.9 | - | - |
| 27 | - | - | - | - | 19.0 (5.3) | 71.9 | - | - |
| 28 | - | - | - | - | 19.0 (3.7) | 80.7 | - | - |

Mean: Mean tick count; Efficacy: Percent efficacy compared to untreated control. Values are shown for the control group and the acaricide treated group in brackets.

**Supplementary Table 7. Therapeutic and persistent efficacy of 1% flumethrin pour-on on goats and 2% flumethrin spray-on on cattle against *Amblyomma variegatum* in the confirmatory studies (nos. 392 & 393)**

| **Day** |  | **Control** | **Flumethrin spray-on (cattle)** | | **Control** | **Flumethrin pour-on  (goats)** | |
| --- | --- | --- | --- | --- | --- | --- | --- |
|  | **Timepoint** | **Mean** | **Mean** | **Efficacy** | **Mean** | **Mean** | **Efficacy** |
| -2 | Tick Challenge | | | | | | |
| 2 | 48 h post acaricide | 17.5 | 0.2 | 99.0 | 14.8 | 9.8 | 33.7 |
| 3 | 72 h post acaricide | 18.0 | 0.0 | 100 | 14.3 | 3.8 | 73.3 |
| 4 | 96 h post acaricide | 18.0 | 0.0 | 100 | 14.2 | 0.3 | 97.6 |
| 14 | Tick Challenge | | | | | | |
| 16 | 48 h post infestation | 28.5 | 17.7 | 38.0 | 19.3 | 2.8 | 85.3 |
| 17 | 72 h post infestation | 28.7 | 13.5 | 52.9 | 18.5 | 1.7 | 91.0 |
| 18 | 96 h post infestation | 29.0 | 13.5 | 53.4 | 18.5 | 0.3 | 98.2 |
| 25 |  | Acaricide administration | | | - | - | - |
| 28 | Tick Challenge | | | | | | |
| 30 | 48 h post infestation | 26.5 | 3.2 | 88.1 | 18.5 | 3.3 | 82.0 |
| 31 | 72 h post infestation | 24.8 | 0.5 | 98.0 | 18.2 | 1.2 | 93.6 |
| 32 | 96 h post infestation | 26.2 | 0.0 | 100 | 18.3 | 0.8 | 95.5 |
| 35 |  | Acaricide administration | | | - | - | - |
| 42 | Tick Challenge | | | | | | |
| 44 | 48 h post infestation | 22.5 | 9.8 | 56.3 | 19.2 | 5.0 | 73.9 |
| 45 | 72 h post infestation | 22.5 | 8.2 | 63.7 | 19.2 | 3.3 | 82.6 |
| 46 | 96 h post infestation | 23.2 | 7.2 | 69.1 | 19.8 | 2.5 | 87.4 |
| 56 | Tick Challenge | | | | | | |
| 58 | 48 h post infestation | - | - | - | 19.6 | 8.0 | 59.2 |
| 59 | 72 h post infestation | - | - | - | 19.6 | 6.0 | 69.4 |
| 60 | 96 h post infestation | - | - | - | 20.0 | 6.0 | 70.0 |

Mean: Mean tick count; Efficacy: Percent efficacy compared to untreated control

**Supplementary Table 8. Therapeutic and persistent efficacy of pour-on formulations; 0.9% fipronil and 0.5% abamectin and 1% fipronil against *Rhipicephalus appendiculatus* ticks in the exploratory study (no. 389)**

| **Day** | **Timepoint** | **Control** | **Fipronil and abamectin** | | **Fipronil** | |
| --- | --- | --- | --- | --- | --- | --- |
|  |  | **Mean** | **Mean** | **Efficacy** | **Mean** | **Efficacy** |
| 1 | 24 h post acaricide | 40.7 | 34.0 | 16.4 | 0.0 | 100 |
| 2 | 48 h post acaricide | 56.3 | 0.3 | 99.4 | 0.0 | 100 |
| 16 | 48 h post infestation | 41.0 | 1.0 | 97.6 | 2.0 | 95.1 |
| 30 | 48 h post infestation | 36.3 | 1.7 | 95.4 | 9.0 | 75.2 |
| 58 | 48 h post infestation | 17.0 | 15.0 | 11.8 | 7.7 | 54.9 |
| 65 | 24 h post infestation | 34.3 | 18.0 | 47.6 | 18.7 | 45.6 |
| 66 | 48 h post infestation | 28.7 | 24.7 | 14.0 | 22.3 | 22.1 |

Mean: Mean tick count; Efficacy: Percent efficacy compared to untreated control

**Supplementary Table 9. Therapeutic efficacy of spray-on formulations; flumethrin dip and spray, amitraz, cymiazole and cypermethrin and chlorfenvinphos and alfamethrin against *Rhipicephalus appendiculatus* ticks in the exploratory study (no. 389)**

| **Timepoint** | **Active Ingredient** | **Control** | **Acaricide** | |
| --- | --- | --- | --- | --- |
|  |  | **Mean** | **Mean** | **Efficacy** |
| 24 h post acaricide | Flumethrin | 40.7 | 0.0 | 100 |
|  | Amitraz | 41.0 | 0.0 | 100 |
|  | Cymiazole and cypermethrin | 36.3 | 3.0 | 91.7 |
|  | Chlorfenvinphos and alfamethrin | 17.0 | 11.3 | 33.3 |
|  | Amitraz | 34.3 | 0.0 | 100 |

Mean: Mean tick count; Efficacy: Percent efficacy compared to untreated control

**Supplementary Table 10. Therapeutic and persistent efficacy of fipronil and abamectin against *Rhipicephalus appendiculatus* ticks in the confirmatory study (no. 390)**

|  |  | **Control** | **Fipronil and abamectin** | |
| --- | --- | --- | --- | --- |
| **Day** | **Timepoint** | **Mean** | **Mean** | **Efficacy** |
| 2 | 48 h post acaricide | 48.3 | 27.8 | 42.4 |
| 3 | 72 h post acaricide | 54.7 | 1.8 | 96.6 |
| 16 | 48 h post infestation | 44.7 | 3.2 | 92.9 |
| 17 | 72 h post infestation | 46.7 | 0.2 | 99.6 |
| 30 | 48 h post infestation | 47.0 | 14.8 | 68.4 |
| 31 | 72 h post infestation | 43.3 | 4.0 | 90.8 |
| 44 | 48 h post infestation | 37.2 | 19.8 | 46.6 |
| 45 | 72 h post infestation | 32.5 | 9.0 | 72.3 |

Mean: Mean tick count; Efficacy: Percent efficacy compared to untreated control
